# Reliability of triggering the stop process is related to prefrontal-subthalamic hyperdirect pathway recruitment

**DOI:** 10.1162/imag_a_00454

**Published:** 2025-01-24

**Authors:** Alexandra Sebastian, Birte U. Forstmann, Dora Matzke

**Affiliations:** Department of Psychiatry and Psychotherapy, University Medical Center of the Johannes Gutenberg University Mainz, Mainz, Germany; Leibniz Institute for Resilience Research, Mainz, Germany; Integrative Model-based Cognitive Neuroscience Research Unit, University of Amsterdam, Amsterdam, The Netherlands; Psychological Methods, Department of Psychology, University of Amsterdam, Amsterdam, Netherlands

**Keywords:** Bayesian modelling, response inhibition, stop-signal task, substantia nigra, subthalamic nucleus, trigger failure

## Abstract

The estimated latency of the unobservable stop response, the so-called stop-signal reaction time (SSRT), has been the established measure of performance in the stop-signal task. While it is currently debated whether SSRT is a suitable marker of inhibition performance, other markers such as the reliability of triggering the stop process (“stop trigger failures”) are coming into focus. In the present study, we elucidated the mechanisms associated with trigger failures using a model-based neuroscience approach by means of functional magnetic resonance imaging for the first time. To this end, we used a large, open-access fMRI data set to investigate the relationship between the probability of trigger failures and fMRI signal change in a stop-signal task in healthy adults (n = 113). Stop trigger failures were associated with less activity in the substantia nigra during unsuccessful stopping and with less activity in the subthalamic nucleus (STN) region during successful inhibition. Although stop trigger failures strongly correlated with SSRT, we found only little evidence for a correlation between SSRT and stopping-related fMRI signal. Thus, in particular, the reliability of the stop process and not its estimated latency depends on the recruitment of key nodes within the prefrontal-subthalamic hyperdirect pathway. More specifically, stop trigger failures may be linked to inadequate substantia nigra innervation at the neural network level. As current evidence suggests that the hyperdirect pathway is engaged by the processing of salient stimuli, deficiencies in assessing the relevance of the stop signal may represent a phenotype associated with a propensity to trigger the stop process unreliably.

## 1 Introduction

Constantly changing environmental conditions and internal states frequently require interruption of ongoing actions in order to maintain goal-oriented behavior. This can be vital, for example, when crossing a road and noticing an approaching car. Response inhibition refers to such suppression or cancelation of an inappropriate behavior and is thus a key cognitive control function (Bari & Robbins, 2013;Miyake et al., 2000;Nigg, 2000;Stahl et al., 2014). According to recent meta-analyses, response inhibition engages a right-lateralized neural network, which includes the right inferior frontal gyrus (IFG), the pre-supplemental motor area (pre-SMA), and subcortically the striatum, the subthalamic nucleus (STN), and the substantia nigra (SN) (Guo et al., 2018;Isherwood et al., 2021).

A prominent task to study response inhibition is the stop-signal task (Logan & Cowan, 1984). It requires participants to perform a two-choice response time (RT) “go” task. In a small proportion of trials, a stop signal occurs shortly after the choice “go” stimulus, instructing participants to cancel their prepared or even initiated response. Participants typically successfully inhibit their response with short intervals between the presentation of the go stimulus and the stop signal (i.e., stop-signal delay; SSD), but not for longer SSDs. Stop-signal performance can be formalized as a race between two independent processes, a go and a stop process. These processes are triggered by the go stimulus and stop signal, respectively. The respective speed of the go and the stop processes as well as the SSD determine the outcome of the race: The response is successfully inhibited if the stop process wins the race and finishes first; otherwise, the response is erroneously executed (Logan & Cowan, 1984;Logan et al., 2014). While the duration of the go response process can be measured directly using go reaction time (RT) on go trials, the stop process itself is unobservable. By assuming that the underlying go RT distributions for trials with and without stop signal are the same, the mean duration for the stop process (i.e., stop-signal RT or SSRT) can be estimated nonparametrically, providing a summary measure of stopping capacity (e.g.,Band et al., 2003;Matzke et al., 2018;Verbruggen et al., 2019). Increased SSRT has been observed in various neuropsychiatric disorders, including Parkinson’s disease (Obeso et al., 2011), attention deficit/hyperactivity disorder (ADHD) (Lijffijt et al., 2005;Lipszyc & Schachar, 2010), substance use and addiction (Chowdhury et al., 2017;Ersche et al., 2012;Liu et al., 2019;Smith et al., 2014), schizophrenia (Lipszyc & Schachar, 2010;Yu et al., 2019), frontotemporal lobar degeneration, and progressive supranuclear palsy (Murley et al., 2020), and is typically considered as a reflection of impulse control deficits. Which processes SSRT depicts and to what extent it actually reflects inhibition performance is, however, the subject of current discussions (e.g.,Diesburg & Wessel, 2021;Huster et al., 2021;Jana et al., 2020). Accordingly, a reconceptualization of inhibition measures and associated neuronal markers is warranted (see also,Choo et al., 2022;Doekemeijer et al., 2021;Skippen et al., 2019).

Other markers that can reflect deficiencies in stop-signal performance besides mean SSRT include more variable SSRT distributions, a higher proportion of slow SSRTs, and the failure of initiating the stop process altogether, that is, stop trigger failures (Sebastian et al., 2018). Failing to trigger the stop process may result from not detecting the stop signal or translating it into an internal stop command (Band et al., 2003) and can be linked to mind wandering (Jana & Aron, 2022). In that respect, stop trigger failures can best be conceptualized as an expression of attentional lapses.

The complete horse-race model allows the estimation of the entire distribution of SSRTs (Colonius, 1990;de Jong et al., 1990;Logan et al., 2014). SSRT distributions can be estimated hierarchically using a Bayesian parametric approach based on the assumption that go RTs and SSRTs follow an ex-Gaussian distribution (Matzke et al., 2013; for software, seeHeathcote et al., 2019). A recent model additionally accounts for failures to trigger the go and the stop processes and corrects SSRT estimates for the resulting bias (Matzke, Love, et al., 2017;Matzke et al., 2019). Increased stop trigger failure rates have been reported in several psychiatric disorders, including ADHD (Weigard et al., 2019), alcohol use disorder (Wang et al., 2023), schizophrenia (Matzke, Hughes, et al., 2017), and post-traumatic stress disorder (Swick & Ashley, 2020). These findings suggest that rather than having difficulties in implementing inhibitory control, inhibitory deficits in these disorders are likely due to difficulties in consistently initiating the inhibitory process, possibly due to attentional deficits. First psychophysiological findings from studies using electroencephalography (EEG) recordings underline the notion of the attentional account of inhibitory deficits (Choo et al., 2022;Matzke, Hughes, et al., 2017;Skippen et al., 2020). Since stop trigger failure rate was strongly increased in right IFG lesion patients in a recent study (Choo et al., 2022), the right IFG may play an important role in the reliability of triggering the stop process.

Taken together, recent advances in the field highlight the importance of trigger failures in the stopping process. It has been acknowledged that the reliability of triggering the stop process is an important measure for individual differences in stop-signal performance next to differences in SSRT latency or variability (Sebastian et al., 2018). Not accounting for stop trigger failures has been shown to bias estimates of stopping latencies (i.e., SSRT is overestimated) (Doekemeijer et al., 2021;Jana et al., 2020;Matzke, Love, et al., 2017;Skippen et al., 2019). Furthermore, the reliability to trigger the stop process has been suggested to be a better predictor of impulsivity than SSRT (Skippen et al., 2019). The underlying mechanisms and neural underpinnings are, however, insufficiently understood to date. We, therefore, investigated the mechanisms associated with the reliability to trigger the stop process in the present study. As the stop process is not initiated in the case of trigger failures, we expected a negative correlation between the reliability of triggering the stop process and the recruitment of the neural stopping network. We, therefore, focused primarily on the core regions of the fronto-basal ganglia network that have been repeatedly and consistently implicated in response inhibition in meta-analyses and review articles, such as right IFG, pre-SMA, striatum, STN, and SN (Aron, 2011;Aron et al., 2016;Cai et al., 2014;Cieslik et al., 2015;Diesburg & Wessel, 2021;Guo et al., 2018;Isherwood et al., 2021). To this end, we used for the first time fMRI to further elucidate the neural underpinnings associated with stop trigger failures using a model-based neuroscience approach (Sebastian et al., 2018) in a large openly available fMRI data set comprising healthy adults (Gorgolewski et al., 2017;Poldrack et al., 2016).

## 2 Material And Methods

The data set (accession number ds000030, revision 1.0.5) was obtained from the OpenfMRI database (https://openfmri.org/dataset/ds000030/) (Poldrack et al., 2016). The data have been collected within the Consortium for Neuropsychiatric Phenomics (CNP), which is a large study funded by the NIH Roadmap Initiative that aims to facilitate discovery of the genetic and environmental bases of variation in psychological and neural system phenotypes, to elucidate the mechanisms that link the human genome to complex psychological syndromes, and to foster breakthroughs in the development of novel treatments for neuropsychiatric disorders. The study includes imaging of a large group of healthy individuals from the community (138 participants), as well as samples of individuals diagnosed with schizophrenia (58), bipolar disorder (49), and ADHD (45). The information regarding participants and experimental design is taken fromPoldrack et al. (2016)and is summarized below.

### 2.1 Participants

The participants of the CNP study were recruited by community advertisements from the Los Angeles area and completed extensive neuropsychological testing, in addition to fMRI scanning. All participants gave written informed consent following procedures approved by the Institutional Review Boards at UCLA and the Los Angeles County Department of Mental Health. Participants were screened for neurological disease, history of head injury with loss of consciousness or cognitive sequelae, use of psychoactive medications, substance dependence within the past 6 months, history of major mental illness or ADHD, and current mood or anxiety disorder, contraindications for MRI, and left-handedness. Urinalysis was used to screen for drugs of abuse (cannabis, amphetamine, opioids, cocaine, benzodiazepines) on the day of testing and excluded if results were positive. A portion of this large sample took part in two separate fMRI sessions, which each included 1 h of behavioral testing and a 1-h scan on the same day. Among other things, the fMRI sessions comprised T1-weighted Anatomical MPRAGE and stop-signal task fMRI. A more detailed study description is provided byPoldrack et al. (2016).

In the present study, we included only individuals of the healthy sample. Of these, five participants had to be excluded due to lacking T1 images (sub-10299, sub-10428, sub-10501, sub-10971, sub-11121), four participants were lacking stop-signal fMRI data (sub-10193, sub-10696, sub-10948, sub-11082), two participants were excluded due to artifacts or signal-drop-out in the stop-signal fMRI data (sub-10460,^*^sub-11067), and six participants were excluded due to irregular task performance, such as high omission rate and associated censoring in the slow tail of the go RT distribution (sub-10225), left-skewed go RT distribution (sub-10963), high frequency of anticipatory go responses (<100 ms; sub-10697), extreme stop-respond probability (90%; sub-11122), and violations of the independence assumption of the race model as evidenced by mean go RT < mean stop-respond RT (sub-10269) or decreasing stop-respond RTs as a function of SSD (sub-10448). The final sample, thus, comprised 113 healthy participants (n = 53/ 46.9% female, M = 31.3 years, SD = 8.87, range: 21–50 years).

### 2.2 Experimental design

All participants included in our study completed one run of the stop-signal task within a behavioral test battery outside the scanner and one run during an fMRI session. Our analyses are based only on the latter. Task and procedure are described in detail byPoldrack et al. (2016). The most important information is briefly summarized below.

#### 2.2.1 Stop-signal task

Participants were shown a series of go stimuli (left- and right-wards pointing arrows), to which they were instructed to respond with left and right button presses, respectively (go trials). On a subset of trials (25%), an auditory stop-signal (a 500 Hz tone, duration: 250 ms) was presented shortly after the presentation of the go stimulus (stop trials). Participants were instructed to respond as quickly and accurately as possible on all trials, but to withhold their response on stop trials and were informed that stopping and going were equally important. On stop trials, the SSD was increased after the participant successfully inhibited in response to a stop signal, and decreased after the participant failed to inhibit in response to a stop signal by 50 ms intervals. The SSD values were drawn from two interleaved staircases, resulting in 16 trials from each staircase for a total of 32 stop trials. On the testing day, participants completed one run outside and one while inside of the MR tomograph. In the first experimental run, initial SSD values for staircase 1 and 2 were 250 and 350 ms, respectively. The last SSD value from each staircase was then used as the initial SSD for the fMRI run. This staircase tracking procedure ensured that participants successfully inhibited on approximately 50% of the stop trials.

Each experimental run contained 128 randomly presented trials, 96 of which were go trials and 32 of which were stop trials. All trials were preceded by a 500 ms fixation cross in the center of the screen. Then, each trial began with the presentation of an arrow and ended after 1,000 ms. Jittered null events separated every trial (with a blank screen), with the duration of null events sampled from an exponential distribution ranging from 0.5 to 4 s, with a mean of 1 s (Poldrack et al., 2016).

#### 2.2.2 MRI data acquisition

MRI data were acquired on one of two 3T Siemens Trio tomographs, located at the Ahmanson-Lovelace Brain Mapping Center (Siemens version syngo MR B15) and the Staglin Center for Cognitive Neuroscience (Siemens version syngo MR B17) at UCLA. Functional MRI data were obtained using a T2*-weighted echoplanar imaging (EPI) sequence (slice thickness = 4 mm, 34 slices, TR = 2 s, TE = 30 ms, flip angle = 90°, matrix 64 × 64, FOV = 192 mm, and oblique slice orientation). Additionally, a high-resolution anatomical data set was obtained using a 3D magnetization-prepared rapid acquisition gradient echo (MPRAGE) sequence (TR = 1.9 s, TE = 2.26 ms, FOV = 250 mm, matrix = 256 × 256, sagittal plane, slice thickness = 1 mm, and 176 slices).

Participants participated in two comprehensive scanning sessions (‘A’ and ‘B’) in a counterbalanced fashion. The MPRAGE was obtained in session A, and stop-signal task fMRI was collected in session B (Poldrack et al., 2016).

### 2.3 Statistical analysis

#### 2.3.1 Behavioral data analysis and Bayesian modeling of behavioral data

To model the behavioral stop-signal data, we used the two-runner BEESTS model as specified inMatzke, Love, et al. (2017), andMatzke et al. (2019). Given the very low average error rate (seeTable 1), we removed all error trials, both on go and stop-respond trials, and only modeled RTs of correct responses. We also removed trials with RTs faster than 150 ms. The model assumed a race between a single go runner and a stop runner, both with ex-Gaussian finishing-time distributions, and allowed for the estimation of the probability of failures to trigger the go and the stop runners. Hence, the model assumed eight parameters: µ_go_, σ_go_, and τ_go_to characterize the finishing-time distribution of the go runner (i.e., the go RT distribution); µ_stop_, σ_stop_, and τ_stop_to characterize the finishing-time distribution of the stop runner (i.e., the SSRT distribution); and GF and TF to quantify the probability of go and stop trigger failures, respectively.

**Table 1.** Descriptive statistics of behavioral performance.

	Mean	SD	Minimum	Maximum
Correct go RT (s)	0.487	0.104	0.338	0.862
Stop respond RT (s)	0.429	0.085	0.315	0.752
SSRT (s)	0.169	0.028	0.104	0.228
Accuracy go	0.979	0.028	0.874	1.000
Accuracy stop	0.512	0.069	0.344	0.688
Omissions go	0.007	0.017	0.000	0.104
TF	0.105	0.091	0.025	0.451
GF	0.005	0.013	4.895e-4	0.081

Note: The model-based statistics (i.e., SSRT, TF, and GF) are based on the mean of the posterior distribution of the participant-level parameters. Stop trigger failures (TF) and go failures (GF) are reported on the probability scale.

Formally, the likelihood that the go runner finishes at time*t*and the stop runner has not yet finished (i.e., stop-respond trial) is L_go_(t) = (1-*GF*) [*TF f*(*t*|**θ**_go_) + (1-*TF*)*f*(*t*|**θ_go_**) S(*t*-SSD|**θ**_stop_)], where*f*(*t*|**θ**_go_) is the ex-Gaussian probability-density function of the finishing time distribution of the go runner with parameters**θ**_go_= (µ_go_, σ_go_, τ_go_) and S(*t*|**θ**_stop_) is the ex-Gaussian survival function of the finishing-time distribution of the stop runner with parameters**θ**_stop_= (µ_stop_, σ_stop_, τ_stop_). The probability of successful stopping is given by$p_{stop}\;=GF\;+\left(1-GF\right)[\left(1-TF\right)\int_{SSD}^{\infty }f\left(t-SSD|\theta_{stop}\right)$$S\left(t|\theta_{go}\right)dt]$, where*f*(*t*|**θ**_stop_) is the ex-Gaussian probability-density function of the finishing time distribution of the stop runner and S(*t*|**θ**_go_) is the ex-Gaussian survival function of the finishing-time distribution of the go runner (for details, seeMatzke et al., 2019).

The model parameters were estimated using Bayesian hierarchical methods (e.g.,Gelman & Hill, 2007;Lee, 2011;Matzke & Wagenmakers, 2009;Rouder et al., 2005). Bayesian hierarchical approaches explicitly model the between-subject variability of the participant-level model parameters using population-level distributions. These population-level distributions act as priors that pull or “shrink” the participant-level estimates closer to the group mean to more moderate values. Especially in situations with scarce participant-level data (as in the current study, in which only 32 stop trials per participant are available), shrinkage typically results in less variable and on average more accurate participant-level estimates than single-level estimation.

As shown in the Supplementary Materials (available at:https://osf.io/6rk3j/), we assumed (truncated) normal population-level distributions for all model parameters parametrized in terms of location and scale. As is standard practice in psychometrics and hierarchical modeling, in order to model them with normal population-level distributions, the GF and TF parameters were projected from the probability scale to the real line using a probit transformation so that the transformed parameters are given by Φ^-1^(GF) and Φ^-1^(TF), where Φ is the standard normal cumulative distribution function. The population-level location parameters were assigned (truncated) normal hyper distributions, with location and scale parameters set to weakly informative values. The population-level scale parameters were assigned exponential prior distributions with a rate of one.

The model was fit to the data using Differential-Evolution Markov Chain Monte Carlo (MCMC) sampling (ter Braak, 2006;Turner et al., 2013) as implemented in the Dynamic Models of Choice software (Heathcote et al., 2019). To facilitate convergence, we first fit each participant’s data separately using non-hierarchical Bayesian estimation and used the resulting estimates as start values for the hierarchical sampling routine. We set the number of MCMC chains to 24, that is, three times the number of participant-level model parameters. To reduce autocorrelation, we thinned each MCMC chain and retained only every 30^th^sample drawn from the joint posterior distribution. During the burn-in period, the probability of a migration step was set to 5%, after which only crossover steps were performed until the MCMC chains converged to their stationary distribution. Convergence was assessed using visual inspection and univariate and multivariate proportional scale-reduction factors (all$\hat{R}$< 1.1;Brooks & Gelman, 1998;Gelman & Rubin, 1992). After convergence, we obtained an additional 100 posterior samples per chain; inference was based on this final set of 24 × 100 = 2,400 posterior samples. The posterior distribution of mean SSRT was obtained by computing µ_stop_+ τ_stop_for each MCMC iteration and then collapsing the resulting samples in a single distribution across chains. The full posterior distribution of all population-level location and scale parameters is presented in the Supplementary Materials (Figs. S1 and S2).

We focused on four aspects of the data to evaluate the descriptive accuracy (i.e., goodness-of-fit) of the model: the distribution of go RTs and stop-respond RTs, inhibition functions, median stop-respond RT as a function of SSD, and the difference between stop-respond RT and go RT. As shown in the Supplementary Materials (Figs. S3–S5), the results showed that the model with the present parametrization provided an excellent description of all these aspects of the observed data.

The correlations reported below were computed using the mean of the participant-level posterior distributions as point estimates. To examine the robustness of the correlational results, we also evaluated the correlations using Bayesian plausible values (Ly et al., 2018). As explained in the Supplementary Materials, the plausible-values analysis imposes a very strict standard of evidence as it treats participants as random effects and hence it takes into account uncertainty from generalizing from the sample of participants to the population as well as the uncertainty encapsulated in the posterior distributions resulting from the relatively scare participant-level data.

In order to assess whether deficiencies in triggering the stop process are related to other key behavioral measures, we correlated trigger failure rate with SSRT, go RT, and go omissions using JASP (JASP Team, 2024; JASP Version 0.18.3). Since omission errors were not normally distributed and 73% of the participants had no omission errors, the variable was probit transformed for the correlation analysis.

### 2.4 fMRI analysis

#### 2.4.1 Preprocessing of fMRI data

All data had been preprocessed using FMRIPREP version 0.4.4 (http://fmriprep.readthedocs.io) as available athttps://legacy.openfmri.org/dataset/ds000030/(revision 1.0.5). For details please cf.Gorgolewski et al. (2017). Subsequently, we applied spatial smoothing using SPM12 (www.fil.ion.ucl.ac.uk/spm/software/spm12/) with a Gaussian kernel with FWHM = 6 mm.

#### 2.4.2 Single-subject analysis

A linear regression model (general linear model; GLM) was fitted to the fMRI data of each subject using SPM12. All events were modeled as stick functions at stimulus onset of the target stimulus and convolved with a canonical hemodynamic response function. The model included a high-pass filter with a cutoff period of 128 s to remove drifts or other low-frequency artifacts in the time series. After convolution with a canonical hemodynamic response function, three event types were modeled as regressors of interest: correct go trials, successful stop trials (i.e., no button press following a stop signal), and unsuccessful stop trials (i.e., button press following a stop signal). In addition, the six covariates containing the realignment parameters capturing the participants’ movements during the experiment were included in the model.

According to the race model (Logan & Cowan, 1984), on unsuccessful stop trials, where the go process finishes before the stop process, the go process is on average faster than on successful stop trials. Conversely, on successful stop trials, the go processes are typically slower, allowing the stop process time to “win” the race and inhibit the response. In other words, stopping fails when the go process is faster than average (fast go), whereas it succeeds when the go process is slower than average (slow go) and thus loses the race. Consequently, RTs for unsuccessful stop trials resemble the faster part of the typically broad go RT distribution within the stop-signal task (Matzke et al., 2013;Verbruggen & Logan, 2015). Neuroimaging and animal studies have reported associations between the speed of the go process and the neural signature (e.g.,Aron & Poldrack, 2006;Messel et al., 2019;Schmidt et al., 2013). Thus, contrasting successful or unsuccessful stop trials with go responses could be confounded by differential neural activity associated with fast versus slow go responses. We, therefore, set up a second GLM with the following four event types as regressors of interest: correct fast go trials (i.e., go RT < median individual go RT), correct slow go trials (i.e., go RT ≥ median individual go RT), successful stop trials, and unsuccessful stop trials. In addition, the six covariates containing the realignment parameters capturing the participants’ movements during the experiment were included in the model.

#### 2.4.3 Group analysis

To identify neural underpinnings of response inhibition, we subjected the three event types that had been modeled on the first level (correct go, successful stop, and unsuccessful stop) to a full-factorial model. We assessed neural activation patterns underlying successful inhibition using the contrast “successful stop > correct go.” Neural activation patterns underlying unsuccessful inhibition were assessed using the contrast “unsuccessful stop > correct go.” Significant effects for each condition were assessed using*t*statistics.

The respective group result was thresholded at p < .05 and corrected for multiple comparisons (family wise error (FWE), correction at peak level) and k = 5 contiguous voxels. The SPM anatomy toolbox 2.0 (Eickhoff et al., 2005,^2006^,^2007^) was used to allocate significant clusters of activation to predefined anatomic regions.

### 2.5 Relationship between stop trigger failures, SSRT, and fMRI signal

To assess the relationship between the stop trigger failure parameter, SSRT, and the fMRI data, we first extracted percent signal change (PSC) using the SPM toolbox “rfxplot” (Gläscher, 2009) from regions of interest (ROI) which constitute key regions of the stopping network (Guo et al., 2018;Isherwood et al., 2021). This was done for both GLMs: 1) the GLM with the regressors of interest being (i) correct go (all), (ii) successful stop, (iii) unsuccessful stop, and 2) the GLM with the regressors of interest being (i) correct fast go, (ii) correct slow go, (iii) successful stop, and (iv) unsuccessful stop. The following masks were used: the right IFG pars opercularis from the Harvard–Oxford atlas included in FSL (Desikan et al., 2006); the pre-SMA ROI was provided byBoekel et al. (2017)and was drawn in MNI space by using the coordinates reported by (Johansen-Berg et al., 2004); and the right striatum (STR), right subthalamic nucleus (STN), and right substantia nigra (SN) ROIs from the probabilistic atlas fromKeuken et al. (2014). All probabilistic masks were thresholded at 10%. Using the 1.5-interquartile range method, we checked the data for outliers in the percent signal change for each given ROI, the probit-transformed stop trigger failure parameter, and in the SSRT. There were no outliers in the stop trigger failure parameter and SSRT. Outliers for percent signal change were excluded for each ROI separately. As outliers varied between ROIs, this approach resulted in different sample sizes (Tables 5and6). Subsequently, we computed Bayesian Pearson correlations for contrasts of interest (i.e., unsuccessful stopping: PSC_unsuccessful stop –__correct go_and successful stopping: PSC_successful stop__–__correct go_) with the probit transformed stop trigger failure parameter (TF) and SSRT, respectively, resulting from the model-based analysis. As outlined above, both fast go and unsuccessful stop trials involve a faster than average go process, whereas both slow go and successful stop trials involve a slower than average go process (cf. alsoSchmidt & Berke, 2017). Therefore, such latency-matched contrasts will help to rule out possible reaction time-related effects on the associated neural signatures. We, therefore, also computed Bayesian Pearson correlations for the latency-matched contrasts (i.e., unsuccessful stopping: PSC_unsuccessful stop –__correct go fast_and successful stopping: PSC_successful stop –__correct go slow_) with the probit transformed stop trigger failure parameter (TF) and SSRT, respectively.

Since we expected less signal with increasing stop trigger failure rates, we tested for a negative correlation. As previous findings on correlations with SSRT are heterogenous (e.g.,Aron & Poldrack, 2006;Aron et al., 2007;Boehler et al., 2012;Chao et al., 2009;Isherwood et al., 2023;Sebastian et al., 2017;Sharp et al., 2010), we performed 2-sided tests in this regard. Resulting Bayes Factors were interpreted following the classification byLee and Wagenmakers (2014). Accordingly, for the null and alternative hypothesis, a Bayes factor between 1/3 and 3 is considered “anecdotal”, between 1/3–1/10 and 3–10 is considered “moderate”, between 1/10–1/30 and 10–30 “strong,” between 1/30–1/100 and 30–100 “very strong”, and Bayes factors less than 1/100 or greater than 100 are considered “extreme”. In addition, we ran multiple regression analyses for the contrasts “successful stop > correct go” and “unsuccessful stop > correct go” to test for correlations with the TF parameter and SSRT on a whole-brain level.

We conducted a mediation analysis using JASP to examine the relationship among stop trigger failures (independent variable), SSRT (mediator), and percent signal change in the STN region during successful stopping (i.e., successful stop – go_slow_). Total, direct, and indirect effects were estimated within a regression-based mediation model. To assess the statistical significance of the indirect effect, we employed nonparametric bootstrapping with 10,000 replications. Bias-corrected 95% confidence intervals were calculated. The significance of the effects was determined based on whether the confidence intervals excluded zero, which indicates a statistically significant effect.

## 3 Results

### 3.1 Behavioral results

Table 1summarizes behavioral data. Participants performed accurately as reflected by high accuracy and low omission error rates on go trials. Commission error rate of stop trials was close to 50%, indicating the adherence of the participants to the task rules and the successful operation of the staircase procedure. Stop trigger failures were present in all participants with a wide range of probability of occurrence across participants (2.5–45%).

Results of Bayesian Pearson correlation of stop trigger failures with stop-signal task performance measures are given inTable 2. There was extreme evidence for a positive correlation with SSRT and strong evidence for a positive correlation with go omissions.

**Table 2.** Bayesian Pearson correlation of stop trigger failures with key behavioral measures of stop-signal task.

	Pearson’s r	BF _10_	95% credible interval
Correct go RT (ms)	−0.142	0.357	[−0.315, 0.044]
SSRT (ms)	0.550***	4.069e+7	[0.401, 0.662]
Omissions go	0.284*	11.388	[0.102, 0.440]

Note: *BF_10_> 10, **BF_10_> 30, ***BF_10_> 100.

### 3.2 Imaging results

#### 3.2.1 fMRI results

As shown inFigure 1, successful and unsuccessful stopping as compared to correct go (Tables 3and4, respectively) both elicited prominent brain activity in multiple prefrontal regions, including the IFG/anterior insula, middle frontal gyrus including inferior frontal junction, pre-SMA, as well as in inferior parietal and superior temporal regions. In addition, significant activation was found in striatal and thalamic regions.

**Fig. 1.**
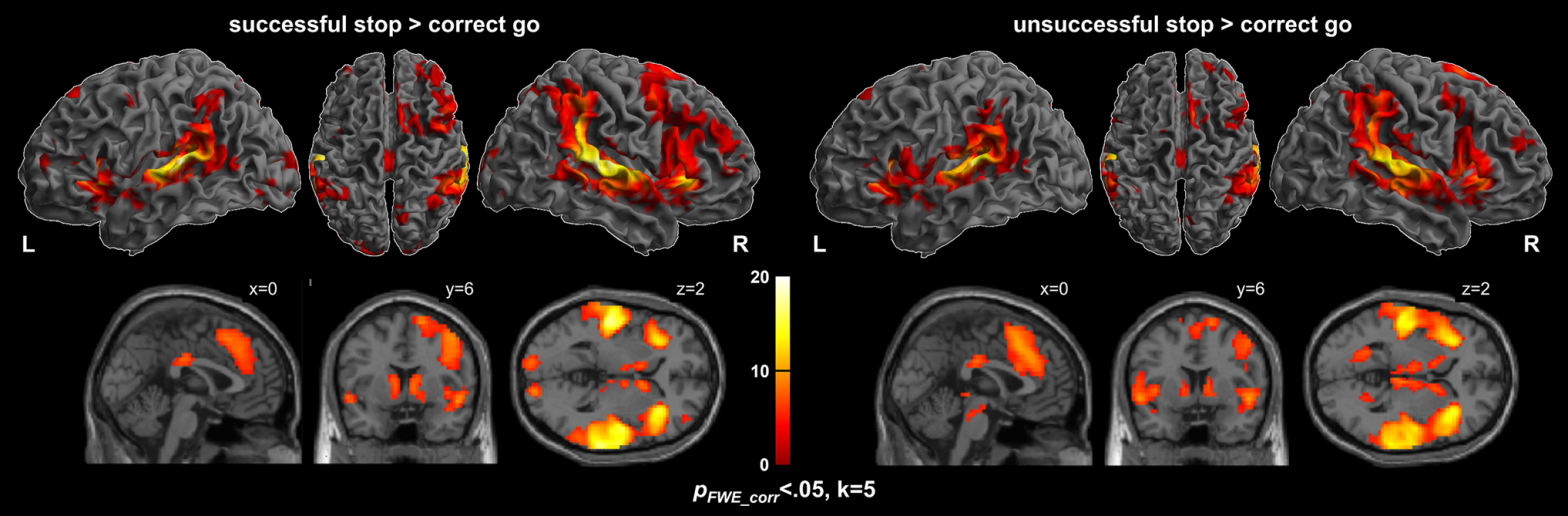
Activation maps for successful inhibition (successful stop > correct go, left panel) and unsuccessful inhibition (unsuccessful stop > correct go, right panel). The color bar represents T scores. p_FWE_< 0.05, k = 5.

**Table 3.** Brain activation underlying successful stopping.

Region	Side	x	y	z	Z	p	k
Superior Temporal Gyrus	R	66	−33	6	Inf	<.001	3,819
Insula Lobe	R	33	24	−2			
SupraMarginal Gyrus	R	63	−42	26			
Superior Temporal Gyrus	R	63	−24	10			
Superior Temporal Gyrus	R	51	−12	2			
Superior Temporal Gyrus	R	45	−27	10			
Inferior Parietal Lobule	R	39	−48	46			
Posterior-Medial Frontal	L	3	18	50			
Posterior-Medial Frontal	R	15	12	66			
IFG (p. Opercularis)	R	48	18	26			
Precentral Gyrus	R	45	3	50			
Superior Temporal Gyrus	L	−63	−27	10	Inf	<.001	1,589
Superior Temporal Gyrus	L	−60	−21	6			
Superior Temporal Gyrus	L	−42	−33	10			
Superior Temporal Gyrus	L	−42	−3	−14			
Insula Lobe	L	−33	21	−6			
Inferior Parietal Lobule	L	−42	−54	46			
Caudate Nucleus	R	9	9	2	Inf	<.001	164
Thalamus	R	9	−9	6			
Thalamus	R	6	−21	−2			
Thalamus	R	15	−27	−2			
Calcarine Gyrus	L	−12	−96	−2	Inf	<.001	153
Middle Occipital Gyrus	L	−18	−99	10			
Inferior Occipital Gyrus	L	−30	−84	−10			
Calcarine Gyrus	R	15	−93	−2	Inf	<.001	99
Fusiform Gyrus	R	30	−78	−10			
Precuneus	R	9	−72	46	Inf	<.001	92
Caudate Nucleus	L	−9	6	6	7.12	<.001	87
Thalamus	L	−9	−6	6			
Thalamus	L	−6	−9	2			
Putamen	L	−21	9	−6			
Middle Cingulate Cortex	R	3	−24	30	7.44	<.001	73
Middle Frontal Gyrus	L	−36	51	14			
Middle Frontal Gyrus	L	−36	51	22	5.31	.002	24
Hippocampus	L	−18	−27	−6	6.19	<.001	19
Cerebellum	L	−18	−78	−34	5.35	.002	16
Precentral Gyrus	L	−45	−3	50	5.48	.001	15
Precuneus	L	−6	−75	42	5.52	.001	9
Cerebellum	L	−27	−72	−30	4.79	.025	5

Note: Local maxima of brain activations during successful stopping (successful stop – correct go) in MNI x-, y-, and z-coordinates with associated Z-score (*p_FWE_*< 0.05, k = 5) and cluster extent in number of voxel (k). Submaxima within a cluster more than 8 mm apart are shown and are indented. R, right; L, left.

**Table 4.** Brain activation underlying unsuccessful stopping.

Region	Side	x	y	z	Z	p	k
Superior Temporal Gyrus	R	66	−33	6	Inf	<.001	2,502
Superior Temporal Gyrus	R	66	−24	10			
Superior Temporal Gyrus	R	51	−24	−6			
Superior Temporal Gyrus	R	45	−27	10			
Superior Temporal Gyrus	R	60	−45	30			
Insula Lobe	R	36	24	2			
Insula Lobe	R	42	21	−2			
Insula Lobe	R	33	21	−10			
IFG (p. Opercularis)	R	54	18	2			
Inferior Parietal Lobule	R	57	−33	54			
Superior Temporal Gyrus	L	−63	−33	10	Inf	<.001	1,793
Superior Temporal Gyrus	L	−63	−24	10			
Superior Temporal Gyrus	L	−48	−18	2			
Superior Temporal Gyrus	L	−45	−6	−10			
Superior Temporal Gyrus	L	−54	6	−6			
Insula Lobe	L	−33	21	6			
Insula Lobe	L	−33	21	−10			
Insula Lobe	L	−42	18	−2			
IFG (p. Opercularis)	L	−57	12	26			
Putamen	L	−18	15	−2			
Posterior-Medial Frontal Lobule	R	15	12	66	Inf	<.001	801
Superior Medial Gyrus	L	3	27	38			
Posterior-Medial Frontal Lobule	L	3	18	50			
Posterior-Medial Frontal Lobule	R	3	15	58			
Middle Cingulate Cortex	R	9	27	34			
Anterior Cingulate Cortex	L	−6	30	26			
Anterior Cingulate Cortex	L	0	18	26			
Diencephalon	L	−9	−27	−10	Inf	<.001	462
Thalamus	R	6	−24	2			
Thalamus	R	9	−12	10			
Thalamus	L	−12	−12	6			
Caudate Nucleus	R	12	6	6			
Calcarine Gyrus	L	−12	−72	10	7.12	<.001	123
Calcarine Gyrus	R	21	−60	6	6.92	<.001	92
Calcarine Gyrus	R	12	−66	10			
Middle Cingulate Cortex	R	3	−21	30	Inf	<.001	90
Precuneus	R	12	−72	42	7.30		58
Cuneus	R	15	−69	38			
Middle Frontal Gyrus	R	24	57	26	6.07	<.001	40
Middle Frontal Gyrus	R	42	45	18	5.57	.001	16
Superior Frontal Gyrus	L	−15	3	70	5.39	.002	7
Inferior Parietal Lobule	L	−39	−51	42	5.26	.003	7
Superior Occipital Gyrus	L	−12	−72	38	5.20	.004	6

Note: Local maxima of brain activations during unsuccessful stopping (unsuccessful stop – correct go) in MNI x-, y-, and z-coordinates with associated Z-score (*p_FWE_*< 0.05, k = 5) and cluster extent in number of voxel (k). Submaxima within a cluster more than 8 mm apart are shown and are indented. R, right; L, left.

#### 3.2.2 Relationship between stop trigger failures and fMRI signal

**Fig. 2.**
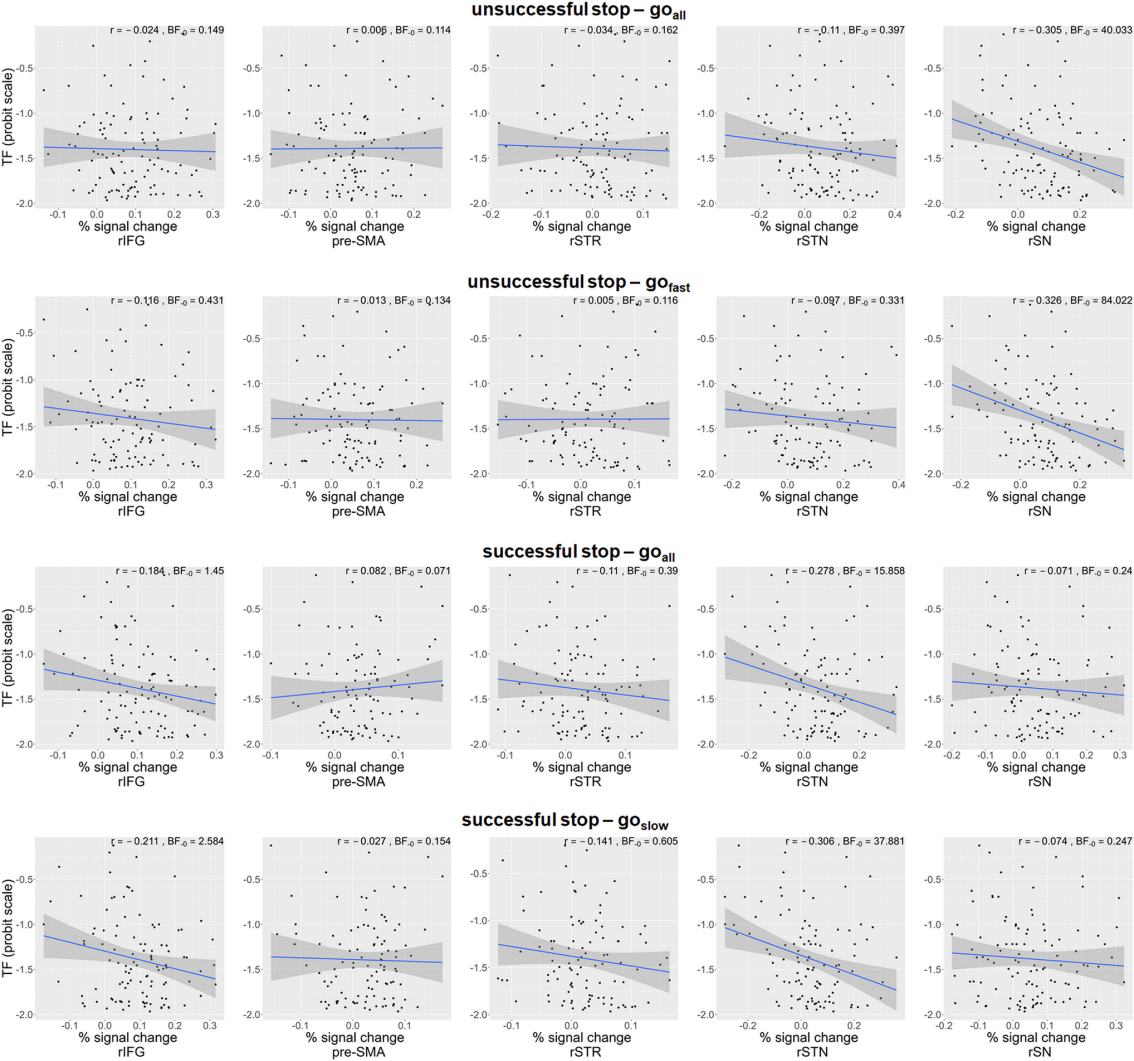
Relationship of precent signal change in regions of interest with stop trigger failures (TF). IFG = inferior frontal gyrus (pars Opercularis); pre-SMA = pre-supplemental motor area; rSTR = right striatum; rSTN = right subthalamic nucleus; rSN = right substantia nigra.

To investigate whether the reliability of triggering the stop process is related to stopping-related brain activity, we first correlated fMRI signal in key-regions of the stopping network associated with successful and unsuccessful stopping with the trigger failure parameter (Fig. 2;Table 5). As expected, increased stop trigger failure rates were associated with decreased activity in key regions of the stopping network. More specifically, there was very strong evidence for a negative correlation of stop trigger failures with percent signal change in the right SN during unsuccessful stopping (i.e., PSC_unsuccessful stop –__correct go__all_), that is, greater trigger failure rate was associated with less right SN activity (Fig. 2, top panel). This was particularly the case when only the latency-matched fast go responses were taken into account in this contrast (i.e., PSC_unsuccessful stop –__correct go__fast_,Fig. 2, upper middle panel). There was no evidence for a relationship with fMRI signal in other regions of interest. Furthermore, for the right IFG, striatum, and pre-SMA, there was moderate evidence for no association with fMRI BOLD signal related to unsuccessful stopping. In addition, a higher probability of stop trigger failures was associated with decreased activity in right IFG and STN during successful stopping (i.e., PSC_successful stop –__correct go__all_;Fig. 2, lower middle panel). Evidence for a correlation with right IFG activity was, however, anecdotal only, whereas evidence for a correlation with activity in right STN during successful stopping was strong. This correlation was particularly strong when only the latency-matched slow go responses were taken into account (i.e., PSC_successful stop – correct go slowl_;Fig. 2, bottom panel). In contrast, there was moderate and strong evidence that there is no association between stop trigger failures and right SN and pre-SMA activity during successful stopping, respectively.

**Table 5.** Summary statistics for the correlation between stop trigger failure rate and percent signal change in regions of interest.

	Pearson’s r	BF _-0_	95% credible interval	n	Pearson’s r	BF _-0_	95% credible interval	n
	PSC _unsuccessful stop – correct go all_				PSC _successful stop – correct go all_			
Right IFG	−0.024	0.149	[−0.229, −0.004]	108	−0.184	1.450	[−0.355, −0.028]	111
Pre-SMA	0.006	0.114	[−0.207, −0.003]	110	0.082	0.071	[−0.169, −0.002]	104
Right STR	−0.034	0.162	[−0.218, 0.153]	109	−0.110	0.390	[−0.290, 0.082]	106
Right STN	−0.110	0.397	[−0.291, −0.009]	112	−0.278*	**15.858**	**[−0.439, −0.095]**	108
Right SN	−0.305**	**40.033**	**[−0.461, −0.122]**	109	−0.071	0.240	[−0.262, −0.006]	110

	PSC _unsuccessful stop – correct go fast_				PSC _successful stop – correct go slow_			
Right IFG	−0.116	0.431	[−0.301, −0.010]	107	−0.211	2.584	[−0.380, −0.041]	109
Pre-SMA	−0.013	0.134	[−0.221, −0.003]	108	−0.027	0.154	[−0.235, −0.004]	104
Right STR	0.005	0.116	[−0.211, −0.003]	107	−0.141	0.605	[−0.325, −0.014]	102
Right STN	−0.097	0.331	[−0.282, −0.008]	110	−0.306**	**37.881**	**[−0.463, −0.121]**	**107**
Right SN	−0.326**	**84.022**	**[−0.480, −0.144]**	**108**	−0.074	0.247	[−0.263, −0.006]	111

Note: Results of Bayesian Pearson correlations are presented for each region of interest. The alternative hypothesis specifies that the correlation is negative (BF_-0_), with a corresponding uniform prior distribution between -1 and 0. Results with BF_-0_≥ 10 are presented in bold. PSC = percent signal change; IFG = inferior frontal gyrus (pars Opercularis); pre-SMA = pre-supplemental motor area; STR = striatum; STN = subthalamic nucleus; SN = substantia nigra; r = Pearsons’s correlation coefficient. *BF_-0_> 10, **BF_-0_> 30.

**Fig. 3.**
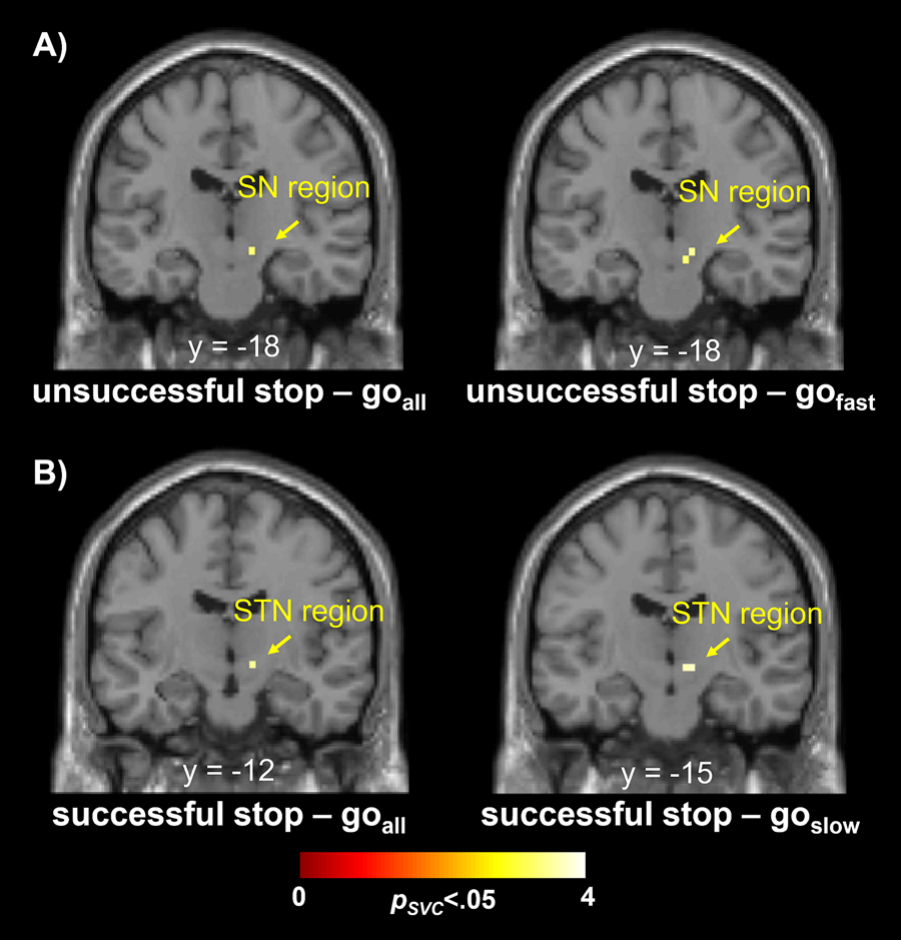
Outcome of multiple regression models for stop trigger failures. On a whole-brain level, (A) during unsuccessful stopping, stop trigger failures were associated with decreased recruitment of the SN region (left: for the comparison with the broad RT distribution,*p_BC_*= .025; right: for the comparison with the latency-matched RT distribution,*p_BC_*= .015). (B) During successful stopping, stop trigger failures were associated with decreased recruitment of the STN region (left: for the comparison with the broad RT distribution,*p_BC_*= .010; right: for the comparison with the latency-matched RT distribution,*p_BC_*= .003).

As the ROI-based approach focuses on specific brain regions only, we additionally exploratively performed correlations on a whole-brain level. The results from the multiple regression analyses were consistent with the results of the region of interest analyses (Fig. 3). Moreover, they revealed no further correlations of the trigger failure parameter with brain activation in other regions. After excluding outliers, probability of stop trigger failures correlated negatively with fMRI signal for the contrast unsuccessful stop > go in the right SN region (unsuccessful stop > go_all_: x = 12, y = -18, z = -10,*Z*= 3.18,*p_SVC_*= .005; unsuccessful stop > go_fast_: x = 12, y = -18, z = -10,*Z*= 3.31,*p_SVC_*= .003). This effect remained significant after controlling for multiple testing using the Bonferroni correction (i.e., after adjusting the significance level (α = 0.05) for five regions of interest/tests:*p_BC_*< .01). For the contrast successful stop > go, probability of stop trigger failures correlated negatively with fMRI signal in the right STN (successful stop > go_all_: x = 12, y = -12, z = -6,*Z*= 3.32,*p_SVC_*= .002; successful stop > go_slow_: x = 12, y = -15, z = -6,*Z*= 3.58,*p_SVC_*= .001). This effect also remained significant after controlling for multiple testing (*p_BC_*< .01). As shown in the Supplemental Materials (Fig. S6), the conservative plausible-values analysis also supported the conclusions from the regions of interest and multiple regression analyses.

#### 3.2.3 Relationship between SSRT and fMRI signal

To investigate whether the trigger failure parameter is specifically associated with neural network activity in the stop-signal task, we also examined the association of SSRT with stopping-related fMRI signal. To this end, we performed the same ROI-based correlational analyses as we did for the trigger failure parameter. As shown inTable 6, the analyses yielded only anecdotal evidence for a negative correlation of SSRT with stopping-related STN signal change. When only the latency-matched slow go responses were taken into account (i.e., PSC_successful stop –__correct go slow_), there was moderate evidence for this negative correlation. Apart from this, there was no evidence for a relationship of SSRT with percent signal change in regions of interest during unsuccessful and successful stopping.

**Table 6.** Summary statistics for the correlation between SSRT and percent signal change in regions of interest.

	Pearson’s r	BF _10_	95% credible interval	n	Pearson’s r	BF _10_	95% credible interval	n
	PSC _unsuccessful stop – correct go all_				PSC _successful stop – correct go all_			
Right IFG	0.006	0.121	[−0.181, 0.192]	108	−0.139	0.339	[−0.314, 0.048]	111
Pre-SMA	0.109	0.225	[−0.079, 0.287]	110	0.029	0.128	[−0.162, 0.217]	104
Right STR	0.044	0.133	[−0.143; 0.228]	109	−0.114	0.236	[−0.294; 0.078]	106
Right STN	−0.137	0.327	[−0.310, 0.050]	112	−0.242	2.766	[−0.407, −0.054]	108
Right SN	−0.124	0.272	[−0.302, 0.0659	109	−0.042	0.131	[−0.225, 0.114]	110

	PSC _unsuccessful stop – correct go fast_				PSC _successful stop – correct go slow_			
Right IFG	−0.006	0.121	[−0.193, 0.182]	107	−0.168	0.535	[−0.341, 0.021]	109
Pre-SMA	0.076	0.162	[−0.113, 0.258]	108	−0.008	0.123	[−0.198, 0.182]	104
Right STR	0.006	0.121	[−0.193, 0.182]	107	−0.094	0.190	[−0.279, 0.102]	102
Right STN	−0.119	0.255	[−0.296, 0.069]	110	−0.257	4.137	[−0.422, −0.070]	107
Right SN	−0.120	0.255	[−0.298, 0.070]	108	−0.033	0.126	[−0.215, 0.153]	111

Note: Results of Bayesian Pearson correlations are presented for each region of interest. PSC = percent signal change; IFG = inferior frontal gyrus (pars Opercularis); pre-SMA = pre-supplemental motor area; STR = striatum; STN = subthalamic nucleus; SN = substantia nigra; r = Pearsons’s correlation coefficient.

The results from the multiple regression analyses were consistent with the results of the region of interest analyses. They also provided no evidence for a correlation between SSRT and brain activation neither in regions of interest nor in other regions. Even after excluding outliers, no significant correlations were revealed.

#### 3.2.4 Relationship between stop trigger failures, SSRT and fMRI signal

Correlational analyses revealed a strong association between stop trigger failures and SSRT, both of which were negatively associated with BOLD signal changes in the STN region within the latency-matched contrast for successful stopping. We conducted a mediation analysis using JASP to examine whether SSRT (mediator) mediates the relationship between stop trigger failures (independent variable) and percent signal change in the STN region during successful stopping (i.e., successful stop – go_slow_).

The total effect of stop trigger failures on stopping-related STN signal change was significant (*b*= -0.087, SE = 0.026, 95% confidence interval [-0.145, -0.026], p <.001), indicating a robust association. However, the indirect effect of stop trigger failures on stopping-related STN signal change via SSRT was not significant (*b*= -0.020, SE = 0.018, 95% confidence interval [-0.057, 0.009], p = .261), suggesting that SSRT does not mediate this relationship. The direct effect of stop trigger failures on STN signal change during successful stopping was significant based on the p-value (p = .033), while the 95% confidence interval included zero (*b*= -0.067, SE = 0.031, 95% confidence interval [-0.132, 0.005]), highlighting some uncertainty in this specific estimate.

## 4 Discussion

While the stopping-specific validity of the SSRT measure is increasingly questioned, stop trigger failures are coming into focus, even though the underlying mechanisms are still largely unknown. It is now acknowledged that the reliability of triggering the stop process is an important measure for individual differences in stop-signal performance (e.g.,Doekemeijer et al., 2021;Sebastian et al., 2018). In the present study, we used for the first time fMRI to further elucidate the neural signature associated with stop trigger failures using a model-based neuroscience approach in a large sample of healthy participants. At the behavioral level, a higher stop trigger failure rate was clearly associated with longer stopping latencies (i.e., SSRT) and increased go omissions, whereas go RT was unaffected by stop trigger failure rate. Correlations with imaging data showed that individuals with a higher propensity for stop trigger failures exhibited stopping-related decreased activity in a fronto-basal ganglia network. More precisely, a higher rate of stop trigger failures was associated with less SN activity during unsuccessful stopping and with less activity in right STN region during successful stopping. These associations were even more pronounced when only latency-matched go responses were considered for the respective contrasts, thereby controlling for potential confounding from neural activity related to response speed. We only found inconclusive evidence for a relationship with right IFG activity during successful stopping. Of note, SSRT was weakly related, if at all, to fMRI BOLD signal in key regions of the neural stopping network highlighting the specificity of the neural correlates of stop trigger failures.

Our imaging findings suggest that trigger failures are related to key regions of fronto-basal ganglia networks—particularly the subcortical nodes—that are known as the indirect and hyperdirect pathways. While the multisynaptic indirect pathway projects from cortical regions to the striatum and downstream to globus pallidus, STN and SN to inhibit motor output, the monosynaptic hyperdirect pathway (Nambu et al., 2002) is thought to circumvent striatum and to directly project from cortex to STN (for a recent review, seeDiesburg & Wessel, 2021). It has long been suggested that this hyperdirect pathway underlies successful stopping (Aron & Poldrack, 2006;Aron et al., 2007). In line with that view, there is meta-analytic neuroimaging evidence that successful stopping engages a right-lateralized network including right IFG, STN and SN (Guo et al., 2018; but seeIsherwood et al., 2021for deviating results). In addition, there is compelling neurophysiological evidence that such a prefrontal-subthalamic hyperdirect pathway is present in humans and mediates rapid stopping (Chen et al., 2020). Recently, however, the role of the prefrontal-subthalamic hyperdirect pathway is being discussed anew. In contrast to other findings, unsuccessful stopping compared to successful stopping and correct go responses were associated with increased STN activity in studies using high-resolution 7T fMRI (Hollander et al., 2017;Isherwood et al., 2023;Miletić et al., 2020). Furthermore, the STN and right IFG were found to be involved not only in the detection of stop signals, but also in response to salient signals in general so that the STN and the hyperdirect pathway have been linked to surprise and unexpectedness (Aron et al., 2016;Fife et al., 2017;Sebastian et al., 2021;Wessel & Aron, 2017). Moreover,Schmidt et al. (2013)demonstrated in their seminal work that in rats STN responds to stop signals irrespective of whether a response was successfully inhibited or not. Similar findings have been reported in non-human primates as well (Pasquereau & Turner, 2017). Recently, it was also shown in humans that stop-specific neurons in the STN fire shortly after the occurrence of a stop signal, independent of the stopping success (Mosher et al., 2021). Taken together, these findings suggest that it is the processing of a salient stimulus such as the stop stimulus rather than the actual implementation of the stopping process that recruits the hyperdirect pathway. While stop signals seem to engage STN activity irrespective of inhibition success (Isherwood et al., 2023;Miletić et al., 2020;Mosher et al., 2021;Schmidt et al., 2013), SN response was specifically linked to successful stopping (Schmidt et al., 2013). As an output nucleus of the STN, the innervation of STN is followed by glutamatergic innervation of SN in successful stop trials (Schmidt et al., 2013) which ultimately has inhibitory effects on motor output (for review cf.Diesburg & Wessel, 2021). Interestingly, for unsuccessful stopping, for which stop trigger failures are contributory causes, stop trigger failures were associated only with decreased SN activity in the present study. As there was no association between TF and the upstream regions of the indirect or hyperdirect pathway, but rather evidence for the absence of such a correlation, it is reasonable to conclude that SN innervation plays a central role in the reliability of triggering the stop process. In contrast, for successful stopping, we found that increasing stop trigger failure rates were associated with decreased STN activity and to a lesser degree IFG activity. By definition, stop trigger failures can only occur in unsuccessful stop trials, but not in successful stop trials. This suggests that individuals with a high propensity for trigger failures may represent a phenotype with general deficiencies in the salience processing of the stop-signal. This may consequently result in a less reliable triggering of the stop process.

Notably, there was little evidence that SSRT was associated with activity in key regions of the neural stopping network despite the strong correlation with stop trigger failures. Only when only latency-matched go responses were considered for the successful stopping contrast was there moderate evidence for an association between SSRT and STN BOLD signal change. No such association was found when comparing to the broad go RT distribution or in whole-brain multiple regression analyses. This is in line with findings byIsherwood et al. (2023)who also accounted for stop trigger failures in their SSRT estimation. The total effect of stop trigger failures on stopping-related STN signal change within the mediation analyses indicates a robust association. The absence of an indirect effect suggests that the effect of unreliability of triggering the stop process on the neural stopping network is not mediated by the the measure of SSRT. In conjunction with the results of the mediation analysis, this underscores the specificity of the association of stop trigger failures with activity in fronto-basal ganglia networks and emphasizes their importance as a measure of stopping capacity.

The present behavioral findings, that is, the correlations between stop trigger failures and go omissions, provide indirect evidence for an association between consistency in triggering the stop process and attentional processes, since omission errors are typically thought to reflect attentional lapses (Trommer et al., 1988;Wright et al., 2014). This interpretation accords with electrophysiological findings suggesting such a relationship. In that context, stop trigger failures correlated with the latency of the N1 to the stop signal, an ERP component associated with early attentional processes (Matzke, Hughes, et al., 2017;Skippen et al., 2020). The so-called stop-N1 effect, an increased N1-amplitude to successful compared to unsuccessful stop trials, has been linked to increased attentional allocation modulating the probability of successful inhibition and resulting in a faster, automatically driven, stop process (Skippen et al., 2020). Moreover, the presumed top-down potentiation of the impact of the stop signal was attributed to right IFG function (Kenemans, 2015). Given the relationship of N1-latency and stop trigger failures,Skippen et al. (2020)suggest that attentional processes involved in triggering the inhibition process may be more important than the speed of the inhibition process (i.e., SSRT) in explaining individual differences in stopping performance. Consistent with this, patients with right IFG lesions had significantly increased stop trigger failures along with significantly reduced stopping-related β-bursts over frontal cortex (Choo et al., 2022). Such β-bursts have been implicated in action stopping (Diesburg et al., 2021;Enz et al., 2021;Jana et al., 2020). Moreover, frontal β-bursts have been suggested to be particularly involved in initiating the cascade that leads to the sensorimotor cortex via the basal ganglia and thus to successful stopping (Choo et al., 2022;Diesburg et al., 2021). In conjunction with the electrophysiological findings, it seems plausible that reduced attentional allocation impairs the initiation of the stopping cascade, which is reflected in stop trigger failures. However, our imaging findings do not provide substantial evidence for an association with right IFG in that respect. There was a numerical negative correlation between stop trigger failures and the right IFG BOLD signal in our data. However, the evidence for a correlation, which only existed for successful stopping, was only anecdotal. In this respect, our results suggest that individuals who trigger the stop process less reliably may have difficulty in processing the impact and relevance of the stop signal. However, this study cannot provide direct evidence for this notion. Multimodal studies combining high temporal resolution methods with high spatial resolution methods would be particularly helpful to test this assumption and to investigate whether there is a link with early sensory processing or the ventral attentional network (Choo et al., 2022;Corbetta & Shulman, 2002,^2011^).

### 4.1 Limitations

Our results are limited by the comparatively large voxel size and the associated smoothing kernel with a FWHM of 6 mm. Such a smoothing kernel increases the risk that signals originating from the subthalamic nucleus and the substantia nigra will get mixed (Hollander et al., 2015). To address these limitations, we firstly used masks of probabilistic atlases of subcortical nuclei based on ultrahigh field imaging data (Keuken et al., 2014). In addition, we reran the correlation analyses after applying smoothing kernel with an FWHM of 3 mm. Applying a smaller smoothing kernel did not substantially change the results, although the correlation coefficients were slightly lower. It would, therefore, be desirable to verify the present results in an independent data set acquired using ultra-high field 7T fMRI using protocols optimized for studying small, iron-rich subcortical structures (Hollander et al., 2017;Miletić et al., 2020).

### 4.2 Conclusions

In summary, the unreliability in triggering the stop process but not its estimated latency (i.e., SSRT) is reflected in a lower recruitment of the prefrontal-subthalamic hyperdirect pathway, which has recently been shown to be associated with the processing of salient signals. Our findings suggest that stop trigger failures at neural network level may be underpinned by inadequate SN innervation. Furthermore, deficiencies in assessing the relevance of the stop signal may represent a phenotype associated with a propensity to trigger the stop process unreliably. Further studies are needed to corroborate these interpretations and to replicate the present results in datasets that are methodologically optimized to detect BOLD signal change in subcortical basal ganglia nuclei.

## Data and Code Availability

The data are freely available and can be obtained from the OpenfMRI data base. Its accession number is ds000030. The preprocessed fMRI data are available athttps://legacy.openfmri.org/dataset/ds000030/(revision 1.0.5). All subsequent fMRI data analyses were conducted using standard processing scripts in SPM12 (https://www.fil.ion.ucl.ac.uk/spm/software/) and the rfxplot toolbox (http://rfxplot.sourceforge.net/). The generic Dynamic Models of Choice code for fitting the hierarchical BEESTS model is available on the Open Science Framework (https://osf.io/5yeh4/). The specific R-scripts used for model fitting are available athttps://osf.io/6rk3j/. Further inquiries can be directed to the corresponding author Alexandra Sebastian (alexandra.sebastian@unimedizin-mainz.de).

## Author Contributions

Alexandra Sebastian: Conceptualization, Methodology, Formal analysis, Data Curation, Writing—Original Draft, Writing—Review & Editing, Visualization, and Funding acquisition. Birte U. Forstmann: Conceptualization, Methodology, Resources, Writing—Review & Editing, Supervision, and Funding acquisition. Dora Matzke: Conceptualization, Software, Methodology, Resources, Formal analysis, Data Curation, Writing—Original Draft, Writing—Review & Editing, Visualization, Supervision, and Funding acquisition.

## Declaration of Competing Interest

The authors report no competing interests relevant to this manuscript.

## Supplementary Material

Supplementary Material

## Footnotes

* Note that subject sub-10460 was included in the hierarchical model of the behavioral stop-signal data.
