# Supplementary material for: Reliability of triggering the stop process is related to prefrontal-subthalamic hyperdirect pathway recruitment
Source: Imaging Neurosci (Camb). 2025 Jan 24;3:imag_a_00454. doi: 10.1162/imag_a_00454 (PMC12319788; doi:10.1162/imag_a_00454)
Supplement: Supplementary Material [file imag_a_00454-supp.pdf]

Supplementary Materials

Prior Distributions

We fit the data on the second scale. We modeled the parameters of each participant *j, j = 1, ..., 114*, with truncated-normal population-level distributions. The participant-level GF and TF parameters were first projected from the probability scale to the real line with a probit transformation. We used the following prior specification, where the first parameter (M) of the (truncated) normal population-level distribution refers to the location and the second (S) to the scale of the distribution. The lower and upper bounds are shown in square brackets.

$$\mu_{go,j} \sim \text{Truncated Normal}(M\mu_{go}, S\mu_{go})[0, 1],$$

$$\sigma_{go,j} \sim \text{Truncated Normal}(M\sigma_{go}, S\sigma_{go})[0, 1],$$

$$\tau_{go,j} \sim \text{Truncated Normal}(M\tau_{go}, S\tau_{go})[0, 1],$$

$$\mu_{stop,j} \sim \text{Truncated Normal}(M\mu_{stop}, S\mu_{stop})[0, 1],$$

$$\sigma_{stop,j} \sim \text{Truncated Normal}(M\sigma_{stop}, S\sigma_{stop})[0, 1],$$

$$\tau_{stop,j} \sim \text{Truncated Normal}(M\tau_{stop}, S\tau_{stop})[0, 1],$$

$$GF_j \sim \text{Normal}(M_{GF}, S_{GF}),$$

$$TF_j \sim \text{Normal}(M_{TF}, S_{TF}),$$

We assigned (truncated) normal prior distributions to the population-level location parameters:

$$M\mu_{go}, M\sigma_{go}, M\tau_{go}, M\mu_{stop}, M\sigma_{stop}, M\tau_{stop} \sim \text{Truncated Normal}(0.5, 10)[0, 1]$$

$$M_{GF,} M_{TF} \sim \text{Normal}(-1.5, 1)$$

We assigned exponential prior distributions to the population-level scale parameters:

$$S\mu_{go}, S\sigma_{go}, S\tau_{go}, S\mu_{stop}, S\sigma_{stop}, S\tau_{stop}, S_{GF}, S_{TF} \sim \text{Exponential}(1)$$

Posterior Distributions

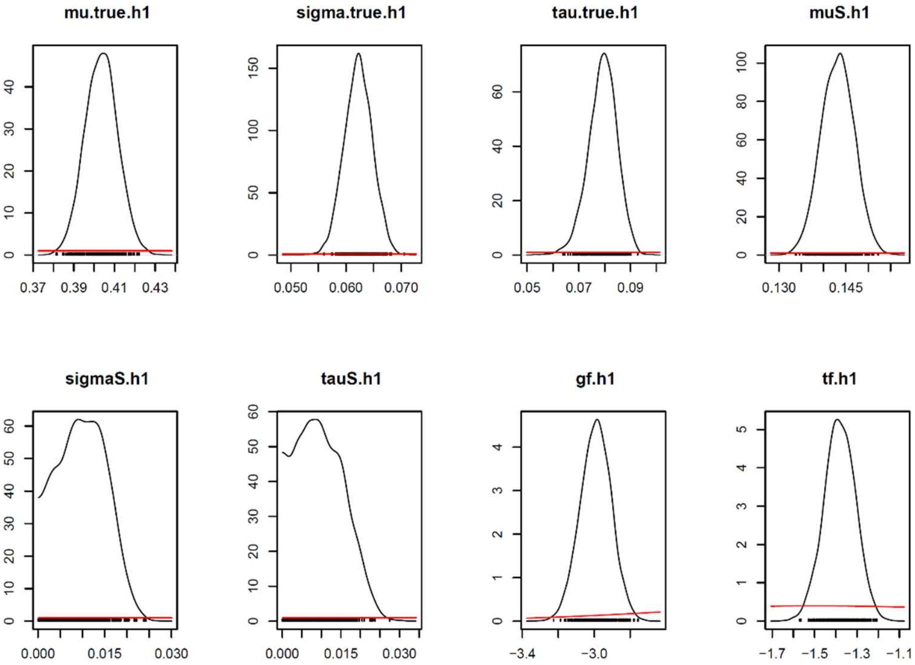

Figure S1. Posterior distribution of the population-level location parameters. The red lines show the prior distributions. mu.true.h1 = Mμ_{go}, sigma.true.h1 = Mσ_{go}, tau.true.h1 = Mτ_{go}, muS.h1 = Mμ_{stop}, sigmaS.h1 = Mσ_{stop}, tauS.h1 = Mτ_{stop}, gf.h1 = M_{GF} (probit scale), and tf.h1 = M_{TF} (probit scale).

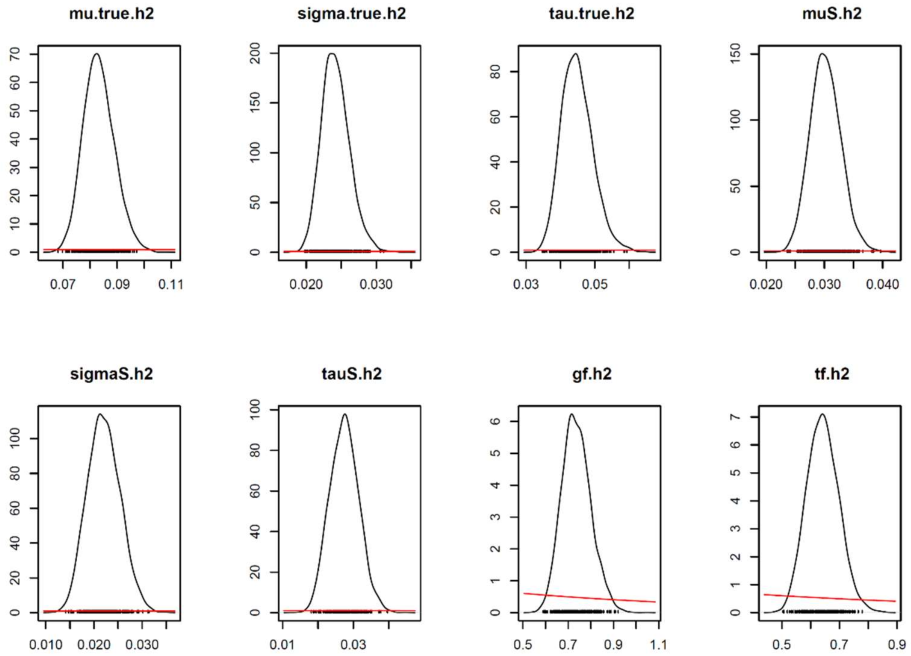

Figure S2. Posterior distribution of the population-level scale parameters. The red lines show the prior distributions. mu.true.h2 = Sμ_{go}, sigma.true.h2 = Sσ_{go}, tau.true.h2 = Sτ_{go}, muS.h2 = Sμ_{stop}, sigmaS.h2 = Sσ_{stop}, tauS.h2 = Sτ_{stop}, gf.h2 = S_{GF} (probit scale), and tf.h2 = S_{TF} (probit scale).

Descriptive Accuracy and Model Assumptions

We used posterior predictive simulations (Gelman et al., 1996) to evaluate the descriptive adequacy of the model. In particular, we compared the observed data to predictions based on the joint posterior distribution of the model parameters. We randomly selected 1000 parameter vectors from the joint posterior distribution of the participant-level model parameters and used these parameter vectors to generate 1000 predicted stop-signal data sets per participant, using the observed stop-signal delays (SSD) and the observed number of go and stop trials. As this procedure relies on the entire joint posterior to generate predictions, it simultaneously accounts for sampling error as well as the uncertainty of the parameter estimates. We focused on three aspects of the data: the distribution of go RTs and stop-respond RTs (Figure S3), the inhibition function (Figure S4, left panel), and median stop-respond RT as a function of SSD (Figure S4, right panel). The predictions closely mimicked the empirical observations, indicating that the model with the present parametrization provided an excellent description of all three aspects the observed data.

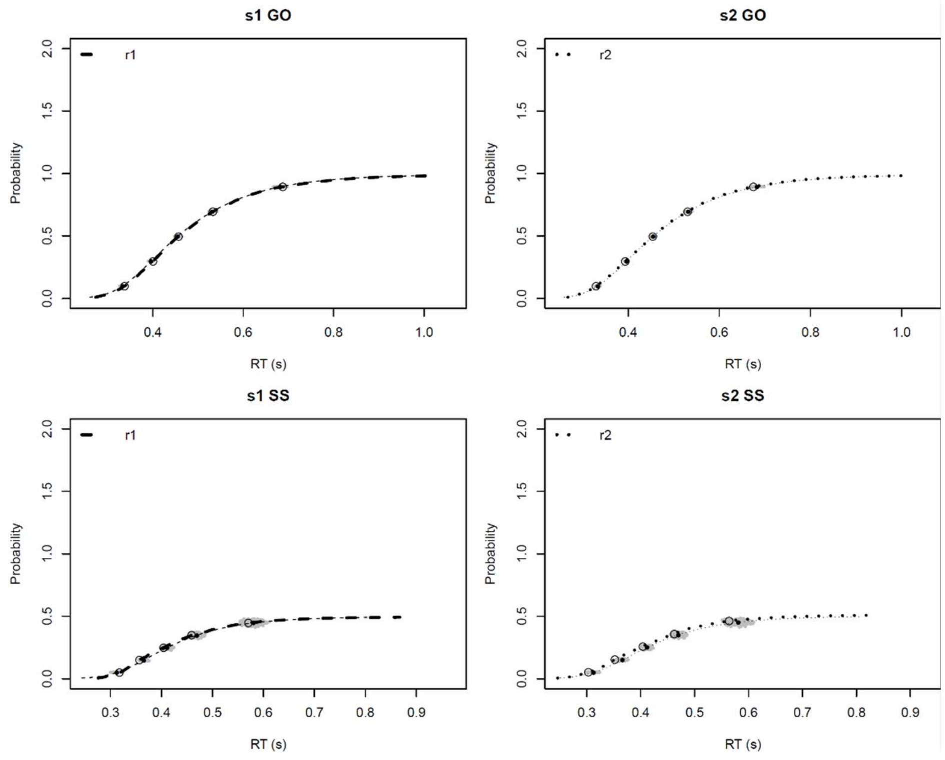

Figure S3. Observed and predicted cumulative distribution function (CDF) of go RTs (upper panels) and stop-respond RTs (lower panels), separately for left (s1; left panels) and right stimuli (s2; right panels). The observed and predicted CDFs were averaged across participants. Stop-respond RTs were collapsed across SSD. Thick dashed and dotted lines show the CDF of the observed "LEFT" (r1) and "RIGHT" (r2) responses, respectively. Black circles show the 10th, 30th, 50th, 70th, and 90th percentile of the distributions. Thin dashed and dotted lines show the CDF of the predicted "LEFT" and "RIGHT" responses, respectively, averaged across the 1000 predictions. For each percentile, the gray clouds show the 1000 predicted percentiles. The upper asymptotes of the CDFs indicate the probability of the

corresponding response. For go RTs, they correspond to one as we only modeled correct responses. For stop-respond RTs they correspond to the response rate on stop trials.

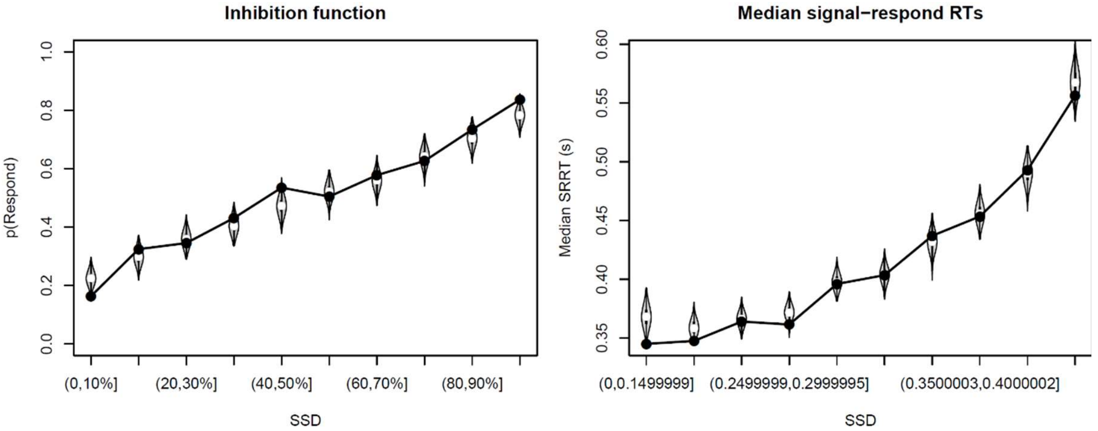

Figure S4. Observed and predicted inhibition function (left panel) and median stop-respond RT as a function of stop-signal delay (SSD; right panel). In the left panel, black bullets (connected by lines) show the observed average response rate on stop trials (P(Respond)) for each SSD category, where the SSD categories were defined in terms of the percentiles of the distribution of SSDs for each participant and then averaged across participants. In the right panel, black bullets (connected by lines) show the observed average median stop-respond RT (SRRT) for each SSD category, where SSD categories were defined by pooling SSDs over participants before calculating the percentiles. The gray violin plots show the distribution of the 1000 average response rates and SRRTs predicted by the model, with the white circles representing the median of the predictions.

The difference between observed stop-respond RT and observed go RT was never positive (Figure S5), suggesting that the assumption of an independent race architecture was not violated on the behavioral level (cf. Bissett et al. 2021).

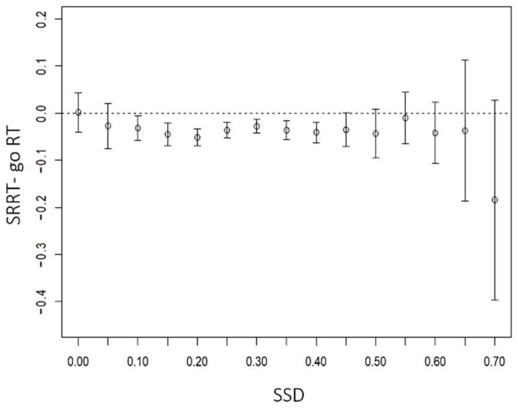

Figure S5. Difference between stop-respond RT (SRRT) and go RT as a function of stop-signal delay (SSD). For details, see Bissett et al. (2021).

Plausible-Values Analysis

For each region of interest, we computed the sample correlation between participants' percentage signal change and each participant-level draw from the posterior distribution of TF (on the probit scale). We then used Ly et al.'s (2018a) analytical solution to compute the posterior distribution of the population correlation corresponding to each sample correlation. The resulting population-level posteriors were averaged to arrive at a single posterior distribution for the population correlation. We used uniform prior distributions between −1 and 1 for the computation of the population-level posteriors. We based inference on the proportion of samples in the posterior distribution of the population correlation above 0, where values close to 0 (for negative correlations) or 1 indicate that the posterior is reliably shifted away from 0. For details, see Ly et al. (2018b). The results are presented in Figure S6. Results for neural activity related to fast and slow RTs are presented in Figures S7 and S8, respectively.

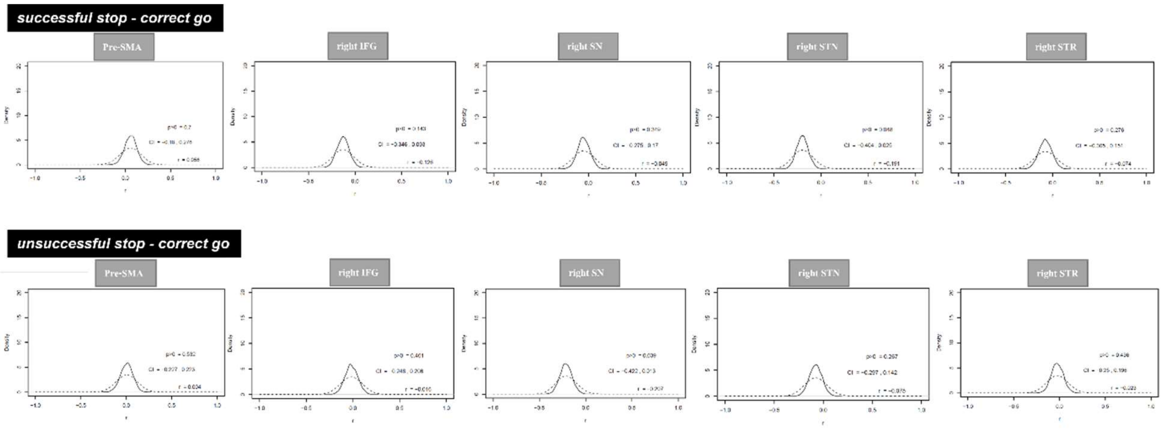

Figure S6. The posterior distribution of the sample (solid) and the population (dashed) correlation between TF and percentage signal change in regions of interest. r = mean of the posterior distribution of the population correlation. CI = 95% credible interval of the posterior distribution of the population correlation. p>0 = proportion of samples in the posterior distribution of the population correlation above 0.

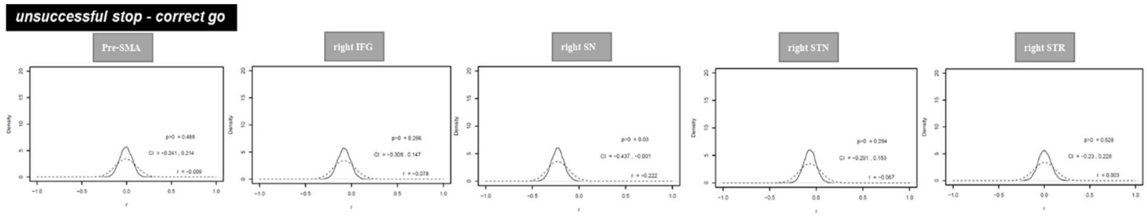

Figure S7. The posterior distribution of the sample (solid) and the population (dashed) correlation between TF and percentage signal change in regions of interest related to fast reaction times. r = mean of the posterior distribution of the population correlation. CI = 95% credible interval of the posterior distribution of the population correlation. p>0 = proportion of samples in the posterior distribution of the population correlation above 0.

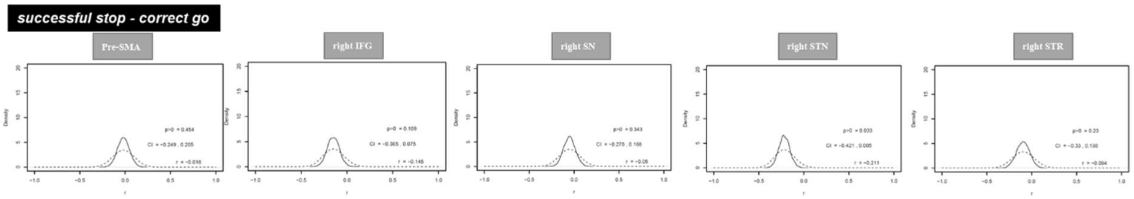

Figure S8. The posterior distribution of the sample (solid) and the population (dashed) correlation between TF and percentage signal change in regions of interest related to slow reaction times. r = mean of the posterior distribution of the population correlation. CI = 95% credible interval of the posterior distribution of the population correlation. p>0 = proportion of samples in the posterior distribution of the population correlation above 0.

References

Bissett, P. G., Jones, H. M., Poldrack, R. A., & Logan, G. D. (2021). Severe violations of independence in response inhibition tasks. *Science Advances, 7*, eabf4355.

Ly, A., Boehm, U., Heathcote, A., Turner, B.M., Forstmann, B., Marsman, M., & Matzke, D. (2018b). A flexible and efficient hierarchical Bayesian approach to the exploration of individual differences in cognitive-model-based neuroscience. In A.A. Moustafa (Ed.), *Computational models of brain and behavior* (pp. 467-480). Wiley Blackwell.

Ly, A., Marsman, M., & Wagenmakers, E.-J. (2018a). Analytic posteriors for Pearson's correlation coefficient. *Statistica Neerlandica, 72*, 4-13.

Gelman, A., Meng, X., & Stern, H. (1996) Posterior predictive assessment of model fitness via realized discrepancies. *Statistica Sinica, 6*, 733–807.
